# Irish GP referral rates and influencing factors

**DOI:** 10.1186/1753-6561-9-S1-A14

**Published:** 2015-01-14

**Authors:** P Gouda, C Mahambo, C Eamonn, L Glynn, G Glynn

**Affiliations:** 1 National University of Ireland, Galway, Ireland; 2 Ark Medical Centre, Unit 1B, Letterkenny Town Centre, Pearce Road, Letterkenny, Co Donegal, Ireland

## Background

General practitioners (GPs) play a key role as the gatekeepers of access to secondary care in Ireland, and indeed in many healthcare systems worldwide [1]. This role has been shown to be crucial in providing cost-effective healthcare delivery. Our study aimed to analyse the GP referral process and the factors by which referral rates may be influenced, particularly those that are unique to the Irish healthcare system.

## Methods

Eighty GPs of the County Sligo General Practitioners’ Society participated between July 2011 and November 2011. For 100 consecutive patient consultation each GP record: patient age, gender, GMS status, and whether or not the patient was referred. In the case of a referral, the GP was asked to specify to what specialty they were referred. Statistical analysis was conducted using PASW Statistics 20.0.

## Results

Of the 7993 consultations, 936 (11.7%) patients were referred to secondary care. There was a wide spectrum of GP referral rates, ranging from 1% to 26%, with a mean average GP referral rate of 11.7% +/- .72%. The emergency department received the greatest proportion of GP referrals (25%). GMS eligibility was found to be associated with referral rates, with 9.7% of GMS eligible patients referred to secondary care compare to 15.3% of GMS ineligible patients, OR 1.67 (95% CI 1.45-1.92). GP gender was also associated with referral rates, with female GPs having a referral rate of 13.2% +/- 6.1 compared to male GPs at 10.4% +/- 6.5 (p = 0.016).

## Conclusions

Our study demonstrates a wide range of GP referral rates. Rather than attempting to standardise referral rates, studies suggest we should strive to reduce inappropriate referral rates [2]. As a result, future studies should aim to measure both the appropriateness of referrals as well as the outcomes of the referral. Although studies of this sort have been conducted in the UK, they have yet to be reproduced in Ireland.
